# Hypercalcemia in Urological Malignancies: A Review

**DOI:** 10.5152/tud.2022.22006

**Published:** 2022-07-01

**Authors:** Gaurav Aggarwal, Sujoy Gupta, Pragyan Khwaunju

**Affiliations:** Department of Uro-Oncology, Tata Medical Center, Kolkata, West Bengal, India

**Keywords:** Hypercalcemia, urological malignancies, humoral hypercalcemia of malignancy, direct osteolytic hypercalcemia

## Abstract

Hypercalcemia is an uncommon occurrence in urological malignancies except for renal cell carcinoma. However, when seen, it is usually associated with advanced disease and both the osteolytic as well as humoral mechanisms may be causative.

Owing to its rarity, hypercalcemia can be easily missed during the initial evaluation of a patient with urologic malignancy.

Our article aims to highlight the mechanisms associated with hypercalcemia in malignancy, in general, and review the available literature on hypercalcemia in urological malignancies. We also aim to discuss the management options in case of such an unusual occurrence in any urological cancer.

**Main Points**

- Since hypercalcemia is a rare occurrence in urological malignancies, it can be easily missed during the initial evaluation of the patient.
- When seen, it is usually associated with advanced disease and both the osteolytic as well as humoral mechanisms may be causative.
- Our article aims to highlight the mechanisms associated with hypercalcemia in malignancy and review the available literature on hypercalcemia in urological malignancies and enlist their respective managements and outcomes.

## Introduction

Hypercalcemia is an uncommon occurrence in urological malignancies except for renal cell carcinoma (RCC). However, when seen, it is usually associated with advanced disease and both the osteolytic as well as humoral mechanisms may be causative. Owing to its rarity, hypercalcemia can be easily missed during the initial evaluation of a patient with urologic malignancy.

We hereby discuss hypercalcemia and its mechanisms of action and also enlist a ­comprehensive review of the literature in hypercalcemia in individual urological malignancies.

## Method

Literature on the current topic was garnered via an extensive PubMed/Medline search employing the MeSH terms—hypercalcemia, urological malignancies, humoral hypercalcemia of malignancy, direct osteolytic hypercalcemia, hypercalcemia in renal cell cancer, hypercalcemia in bladder cancer, hypercalcemia in penile cancer, hypercalcemia in prostate cancer, hypercalcemia in testicular cancer, and hypercalcemia in urological malignancy.

All articles encompassing any of the above MeSH terms, in relation to urological malignancies, were included in the review.

## What Is Hypercalcemia?

Hypercalcemia is defined as serum calcium concentration two standard deviations above the mean, or simply stated, serum calcium levels of more than 10.5 mg/dl.^1,2^

**Corrected Serum Calcium and Role of Albumin in Calcium Homeostasis**

About 45% of calcium in the peripheral blood is bound to albumin; 10% is bound to anions such as phosphate and citrate, and the remaining 45% is the free or ionized calcium. Each g/dL of albumin binds to 0.8 mg/dL of calcium.^1,2^ Alteration of serum albumin level hence will alter the ionized calcium level for the same value of total serum calcium.

It is this ionized calcium that is readily available for activating cellular processes. Therefore serum calcium level needs to be corrected as per the albumin levels of the patient. Corrected serum calcium can be calculated as: CSC = 0.8 × (4 – serum albumin) + serum calcium. Based on the level of corrected calcium level in blood, it can be classified as mild (Ca—10.5-11.9), moderate (Ca—12-13.9), and severe hypercalcemia (Ca > 14). Corrected serum calcium levels of more than 15 mg/dl are considered as a medical emergency and need immediate correction.

## Symptoms of Hypercalcemia

Mild hypercalcemia can itself be asymptomatic or symptomatic associated with lethargy and/or skeletal pain. Moderate to severe hypercalcemia, on the other hand, may cause neurocognitive symptoms, significant volume depletion, acute renal insufficiency, and even death, particularly in severe cases.^1,2^

The common symptoms of hypercalcemia may be classified as:

- Neurological symptoms (lethargy, weakness, confusion, coma).
- Gastrointestinal symptoms (constipation, nausea, anorexia, gastric ulcer, pancreatitis) or
- Renal symptoms (polyuria, nocturia, dehydration, stone).
- Others: Life-threatening arrhythmias or cardiac arrest (with severe hypercalcemia).

## Hypercalcemia in Malignancy

The most common cause of hypercalcemia, per se, is hyperparathyroidism. Hypercalcemia in hyperparathyroidism is usually mild, asymptomatic, and long-standing. Rapidly increasing hypercalcemia should however increase the suspicion of malignancy.

The estimated prevalence of hypercalcemia in malignancy is 1.46-2.74%^2^ and the most common malignancies associated with hypercalcemia are lung carcinoma, multiple myeloma, and RCC. Hypercalcemia is usually associated with an advanced stage of malignancy and is considered a poor prognostic factor.

The incidence of hypercalcemia in different malignancies as seen in the study conducted with electronic health records (EHR) data using the Oncology Services Comprehensive Electronic Records (OSCER) database is shown in Table 1.^3^

## Mechanism of Hypercalcemia in Malignancy

Hypercalcemia in malignancy can be due to several mechanisms. The two most common causes of hypercalcemia in malignancy are humoral hypercalcemia and direct osteolytic hypercalcemia.

- Humoral hypercalcemia of malignancy (HHM):

The release of the mediators by the tumor that causes bone resorption and subsequently increased serum calcium without the tumor directly invading the bony tissue is called HHM.

The term HHM was first proposed by Fuller Albright in 1941^4^ and it specifically refers to parathyroid hormone-related peptide (PTHrP) mediated hypercalcemia. It constitutes about 80% of hypercalcemia in malignancies^5^ and is commonly seen in squamous cell carcinoma of the esophagus, head and neck, lung, cervical cancers as well as in RCC, bladder cancer, breast cancer, endometrial and ovarian cancers.

Parathyroid hormone-related peptide is normally secreted by mesenchymal stem cells. It functions in endocrine, paracrine, autocrine, and intracrine manners and has a role in endochondral bone development, tooth eruption, and mammary gland development. When secreted by tumor cells, it can cause hypercalcemia. Parathyroid hormone and PTHrP are similar molecules, therefore, both are not concurrently elevated unless there are multiple etiologies.

- Direct osteolytic hypercalcemia:

In osteolytic hypercalcemia, there is a direct invasion of bone by the tumor, causing osteolysis and subsequent hypercalcemia.

Local osteolytic hypercalcemia is seen in 20% of malignancies and is commonly seen in multiple myeloma and breast cancers.^6^ It is thought to be due to the release of local cytokines which cause excessive osteoclast activation and bone resorption, mediated through RANK/RANKL complex. Besides these, other less common causes of hypercalcemia of malignancies are extrarenal production of 1,25(OH)2D, ectopic parathyroid production, or associated primary hyperparathyroidism.^7^

## Hypercalcemia in Urological Malignancies

Hypercalcemia is an uncommon occurrence in urological malignancies except for RCC. However, when seen, it is usually associated with advanced disease and both the osteolytic as well as humoral mechanisms may be causative.

Owing to its rarity, hypercalcemia can be easily missed during the initial evaluation of a patient with urologic malignancy. Any patient with advanced urologic malignancy, in particular, with symptoms of lethargy, weakness, or gastrointestinal distress should be evaluated with a suspicion of having hypercalcemia.

**Figure 1.**
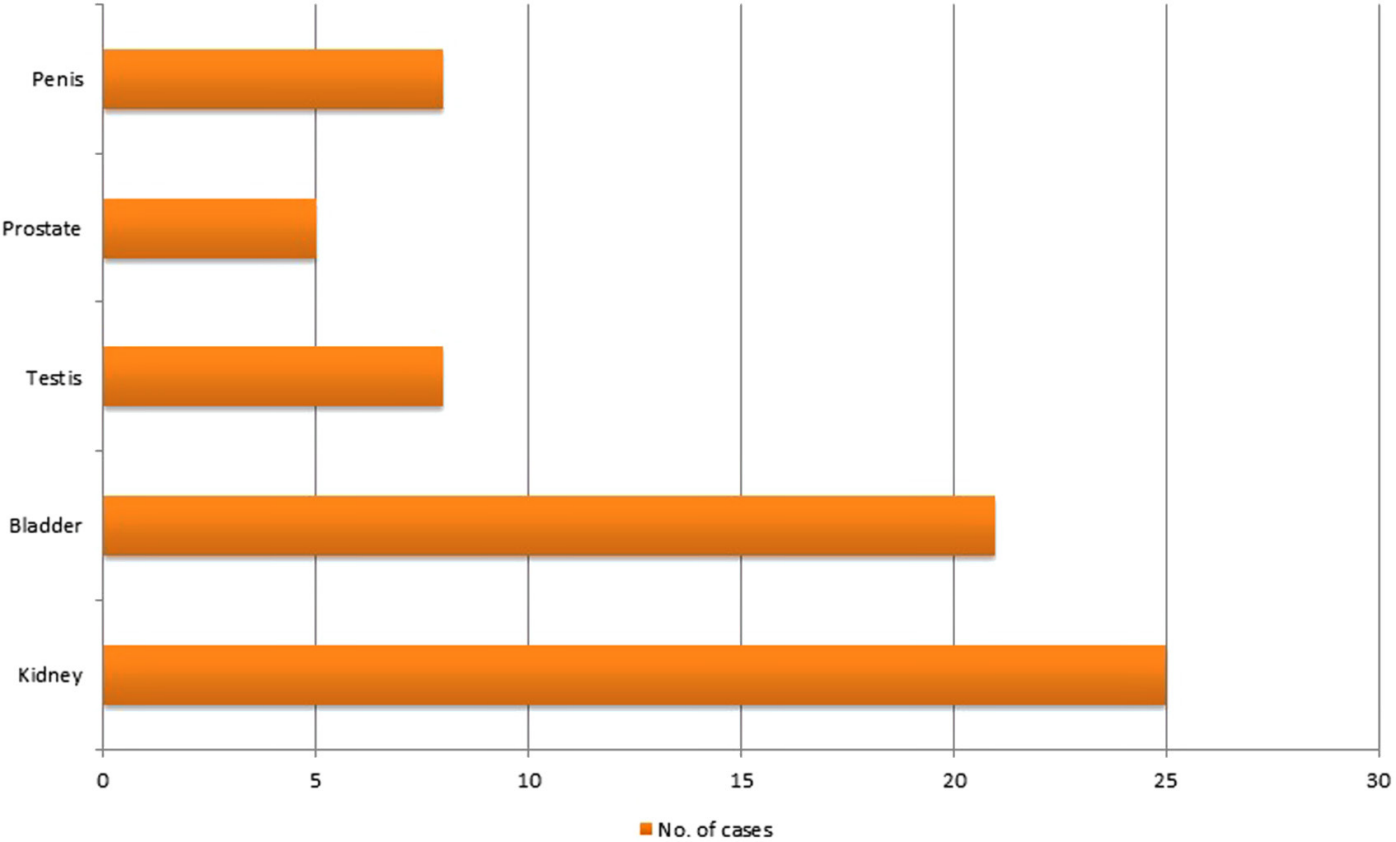
Frequency of cases of Urological malignancies, with hypercalcemia, reported in the literature, to date.

**Figure 2.**
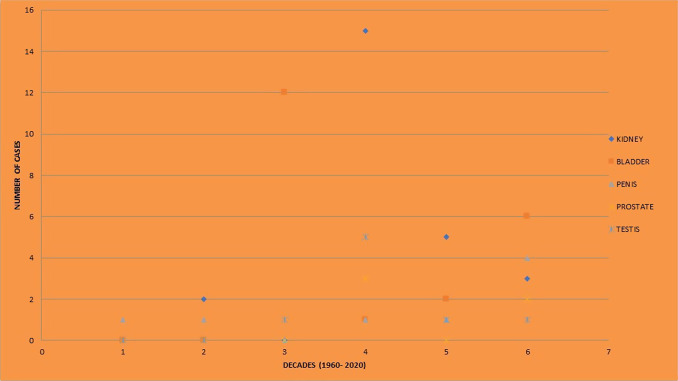
Decade-wise distribution of cases of urological malignancies with hypercalcemia, as reported in the literature.Y Axis: Numbers of cases of an individual urological malignancy with hypercalcemia.X Axis: Decades: 1 = 1960-1970; 2 = 1970-1980; 3 = 1980-1990; 4 = 1990-2000; 5 = 2000-2010; 6 = 2010-2020; 7 = >2020.

Figure 1 depicts the number of cases of Urological malignancies, with hypercalcemia, reported in the literature, to date. Figure 2 depicts the decade-wise distribution of these cases, as reported in the literature.

## Let Us Look at Individual Urological Malignancies Hereafter

### Renal Cell Carcinoma

The most common presentation of RCC is the triad of hematuria (50-60%), abdominal pain (40%), and palpable mass (30%).^8^

Renal cell carcinoma is also the most common urologic malignancy associated with various paraneoplastic syndromes, seen in nearly 30% of patients, namely elevated erythrocyte sedimentation rate (ESR), hypercalcemia, polycythemia, hepatic dysfunction (Stauffer’s syndrome), amyloidosis, hypertension, and fever.^9^ Most of these remit with the treatment of the primary disease.

Approximately 17% of all patients with RCC develop hypercalcemia during the course of their disease.^10^ Humoral hypercalcemia of malignancy is seen to affect 13-20% of these.^11^ Yu Yang et al retrospectively analyzed 464 patients with RCC, 562 cases of prostate cancer, and 647 with bladder cancer from 2005 to 2010 and found that 156 (33.6%) patients of RCC were diagnosed with paraneoplastic syndromes manifesting as anemia, hypertension, fever, polycythemia, Stauffer's syndrome, fatigue, weight loss, anorexia, leukocytosis, and hypercalcemia. Two prostate cancer patients had elevated levels of calcium and blood glucose, respectively. However, no specific clinical presentation in bladder cancer was found to be associated with paraneoplastic syndromes, in their study.^12^

Albright in 1941 postulated that hypercalcemia was because of a humoral factor secreted by the tumor cells.^13^ In the late 1980s, this substance was identified and named as PTHrP. It is seen to be elevated in up to 15% of all patients with RCC. Other humoral factors that have also been associated with hypercalcemia in RCC include IL-6, IL-1, TNFα, transforming growth factor-alpha and beta, and prostaglandins.^13,14^ IL-6 has been shown to activate osteoclastic bone resorption but it is unclear whether this cytokine causes hypercalcemia directly or by the effect of PTHrP. It has been seen to act synergistically when co-expressed with PTHrP. Other causes of hypercalcemia in cases of RCC include medications like premarin, lytic bone lesions, and ectopic PTH secretion.

Elevated corrected calcium concentration may be considered as a potential biomarker for assessing the prognosis of this disease. However, though an association has been found, this has not yet been proven in case of localized RCC. A study by Fahn et al which studied 20 hypercalcemic patients with RCC showed that the survival curves between the hypercalcemic and eucalcemic groups among stages I to III cancer patients showed no statistically significant difference (*P* > .05). The survival curve deteriorated significantly in stage IV cancer patients with humoral hypercalcemia (*P* < .005), with a median survival of 45.0 ± 39.7 days vs. 286.4 ± 27.6 days in eucalcemic patients.^15^ On the other hand a meta-analysis of Mao et al which included a total of 13 studies with 6705 patients has shown that elevated corrected calcium levels predicted poorer overall survival (OS) in RCC (HR = 1.95; 95% CI = 1.67-2.27; *P* < .001). On subgroup analysis that included tumor type, analysis type, cut-off value, and ethnicity, elevated corrected calcium levels were associated with poorer OS for all stages of RCC as well as for patients with metastatic RCC.^16^

The role of nephrectomy in the management of hypercalcemia in metastatic renal carcinoma is not well established. In the era before cytokine therapy, cytoreductive surgery was commonly practiced for hypercalcemia of RCC. Nephrectomy has been shown to temporarily ameliorate hypercalcemia in a subgroup of patients with metastatic renal cancer and hypercalcemia in a study by Walther et al. In their study, a decrease in serum calcium corrected for albumin occurred in 9 of 11 patients at 1 to 4 weeks after nephrectomy and in 7 of 12 patients at 5 to 16 weeks after nephrectomy.^17^ However, with the emergence of targeted therapy for advanced RCC, the role of cytoreductive nephrectomy has become a debated topic. Few studies have shown that sunitinib, an oral multitargeted tyrosine kinase inhibitor, normalizes hypercalcemia in a metastatic RCC thus eliminating the need for surgery in such patients.^18^ Table 2 lists the reports of RCC with hypercalcemia, in the literature, to date.

### Bladder Cancer

Bladder cancer typically presents with macroscopic hematuria. Most bladder cancers are urothelial (transitional cell) malignancies followed by occasionally squamous cell and adenocarcinoma. Hypercalcemia in bladder cancer is not a common occurrence and if present it is usually associated with the bony metastasis or large tumor volume. Very few cases of bladder cancers associated with raised PTHrP have been reported in the literature. The outcome of such malignancies is usually very poor.

Although most cases of urothelial carcinoma associated with paraneoplastic hypercalcemia tend to be high grade and aggressive in nature with a poor prognosis,^28^ few have been reported without any metastasis.^29,30^ Outcome of such tumors is reported as usually good, as hypercalcemia tends to resolve after radical cystectomy.^31^ Serum calcium levels normalize during the immediate postoperative period itself. However, regular serum calcium level monitoring is needed in such patients because the recurrence of hypercalcemia may likely be a surrogate sign of tumor recurrence in the lymph nodes or even bony metastasis.^32^

There is, however, no preferred treatment for hypercalcemia associated with the recurrence of such malignancies. Radiotherapy or chemotherapy may be helpful to ameliorate the symptoms, but these patients are usually poor candidates for chemotherapy. Intravenous hydration, bisphosphonates, calcitonin, and denosumab are the options available to attempt a decrease calcium levels.

Few cases of small cell carcinoma of bladder causing hypercalcemia have also been reported in the literature.^33,34^ Cesar V Reyes reported 2 cases of small cell carcinoma of bladder. In both cases, despite treatment, the disease progressed and patients died. Table 3 lists the literature available on bladder cancer with hypercalcemia.

### Penile Carcinoma

Most penile carcinomas are localized to the prepuce or glans penis. The regional disease is seen in about 13% of cases and distant metastasis is seen in 2.3%. Although squamous cell carcinomas (SCC) are collectively the most common cause of HHM, SCC of the penis rarely presents with hypercalcemia. Like in any other urologic malignancy, hypercalcemia in penile carcinoma tends to be associated with advanced disease and/or bony metastasis.

Hypercalcemia has also been reported without bony metastasis which is attributable to the paraneoplastic syndrome due to the expression of PTHrP.^41^ Penile malignancy with hypercalcemia without bony metastasis was first reported by Anderson et al in 1965. The case was a non-metastatic SCC of penis which was managed with surgical removal of tumor. The serum calcium level returned to normal within 24 hours of removal of the primary penile lesion. However, the level of PTHrP was undocumented in this case.^42^ Malakoff and Schmidt described a patient with metastatic penile carcinoma which was complicated with serious hypercalcemia due to secretion of PTH. Management of the hypercalcemia by oral biphosphonates, hydration, and diuretics showed only a temporary response. There was transient response to furosemide-induced dieresis and he was refractory to treatment with oral inorganic phosphates and mithramycin. Ablation of the primary tumor did not affect his hypercalcemia. His serum calcium stabilized and became normal after external irradiation and parenteral bleomycin therapy. However, this response was only temporary as there was the progression of the tumor.^43^ In another case reported by Trejo et al. hypercalcemia in penile carcinoma was associated with PTHrP secretion. It was managed initially with vigorous hydration and loop diuretics without achieving a satisfactory calcium level. A dose of 4 mg zoledronic acid was therefore infused, resulting in a remarkable response. Normal calcium levels were achieved by 4 weeks, at which time another dose was needed.^44^

There are also few case reports of penile carcinoma presenting with hypercalcemia due to ectopic production of parathyroid hormone. Sardinas et al reported a case of penile carcinoma with hypercalcemia with elevated PTH and low/normal PTHrP. Lesional fluid aspirated during the biopsy contained very high levels of PTH, thus confirming its ectopic source from this metastatic squamous cell neoplasm.^45^

Gandhi et al reported a case of extensive visceral calcification demonstrated on ^99m^Tc-MDP bone scan in patient with carcinoma penis and hypercalcemia of malignancy. These metastatic calcifications are mostly reversible after normalization of calcium metabolism and renal function with the disappearance of the increased uptake on bone scan.^46^

Table 4 lists the literature available on penile cancer with hypercalcemia.

### Prostate Cancer

Prostate cancer is the second most common cancer and the fourth most common cause of cancer death in men. Bony metastasis and associated skeletal events like pathological fractures and cord compressions are not uncommon in metastatic carcinoma of the prostate.^2^

However, hypercalcemia is a rare manifestation of prostate cancer because these metastasis are osteoblastic. In a retrospective study conducted between 2009 and 2013 using the OSCER warehouse of EHR, the rates of malignancy-induced hypercalcemia were the lowest among patients with prostate cancer, ranging from 1.4% to 2.1%.^2^ The exact pathophysiology of hypercalcemia in prostate cancer is unknown although humoral hypercalcemia and osteolytic hypercalcemia both have been proposed.

A study by Iwamura et al suggested that PTHrP may play a significant role in the growth of prostate cancer by acting locally in an autocrine fashion. Their study showed that all prostate cancer cell lines from different sources expressed PTHrP immunoreactivity as well as evidence of DNA synthesis. The cancer cell line derived from bone metastatic lesions secreted significantly greater amounts of PTHrP than did the cell line derived from the metastasis in the brain or in the lymph node. These findings suggest that PTHrP production may confer some advantage on the ability of prostate cancer cells to grow in bone. However, in spite of widespread expression of PTHrP in prostate cancer, hypercalcemia is still not so common probably due to counteraction of other factors like simultaneous secretion of calcitonin.^49^

Some studies have shown that hypercalcemia in prostate cancer is associated with unusual histology, particularly neuroendocrine differentiation of adenocarcinoma. A study by Takashi et al showed that neuroendocrine development as a result of androgen deprivation therapy caused hypercalcemia due to PTHrP secretion, which was lethal. Thus, early detection and treatment of neuroendocrine differentiation would improve prognosis. However, Prostate Specific Antigen (PSA) is not an appropriate marker for detecting neuroendocrine differentiation. Hence periodic monitoring of neuroendocrine markers such as neuron-specific enolase should be considered.^50^ In a case reported by Alhatemi et al. a case of carcinoma of the prostate with hypercalcemia had osteoblastic lesions on the bone scan, hence ruling out the local osteolytic bone destruction theory for hypercalcemia. Furthermore, PTHrP was not significantly elevated in this patient, PTH levels were normal and histopathology showed no evidence of mosaicism or neuroendocrine dedifferentiation. These findings in aggregate tell us that an exact pathophysiologic mechanism leading to hypercalcemia in prostate cancer is still unclear.^51^ There can be an interplay of different factors apart from these which need to be proven by future research.

Table 5 lists the literature available on prostate cancer with hypercalcemia.

### Testicular Tumor

Sixty percent of the testicular tumors are seminomas. Very few cases of seminoma associated with hypercalcemia have been reported, and their association with PTHrP has not been studied in those few reported cases. In contrast to other urologic malignancies with hypercalcemia, testicular tumor with hypercalcemia usually has a good prognosis.^53,54^

Grote et al and Da silva et al reported the case of seminoma with hypercalcemia due to raised 1,25 dihydroxy cholecalciferol. Hypercalcemia resolved after treatment of the primary tumor in these reported cases.^53,54^ MacDiarmid et al in 1995 reported malignant hypercalcemia associated with an extragonadal Non-seminomatous germ cell tumor (NSGCT) that was suspected to be probably due to humoral hypercalcemia after bone scans were negative. Parathyroid hormone-related peptides and vitamin D metabolites were not measured.^55^ Steven Sorscher reported the first case of biochemically confirmed PTHrP-induced hypercalcemia in a germ cell tumor. This was also the second case of an extragonadal nonseminomatous germ-cell tumor associated with hypercalcemia. Parathyroid hormone-related peptide was found to be elevated and PTH suppressed. Abnormal laboratory findings normalized, after treatment of the primary tumor with chemotherapy.^56^ Rodríguez-Gutiérrez et al reported a case of hypercalcemia associated with rise in PTHrP as well as 1,25 dihydroxy cholecalciferol. The patient had presented with a calcium level of 14.7. Calcitonin and hydration with intravenous saline solution at 250 mL/h were started. Despite therapy, calcium lowered only to 13.5 mg/dl, but nevertheless, his mental status improved. The patient received 4 cycles of bleomycin, etoposide, and carboplatin. After the first cycle of chemotherapy, calcium, Lactate dehydrogenase (LDH), and alkaline phosphatase returned to normal values. Both PTHrP and 1,25(OH)2D3 dropped dramatically. At 8 months follow-up, the patient was asymptomatic.^57^

Table 6 lists the literature available on testicular cancer with hypercalcemia.

## Conclusion

Hypercalcemia although generally rare in urologic malignancies may sometimes be seen in advanced cases. Humoral hypercalcemia of malignancy due to raised PTHrP is the most common cause of hypercalcemia followed by osteolytic hypercalcemia. The mainstays of treatment are hydration, bisphosphonates, and calcitonin. Treatment of the primary is necessary to treat as well as to prevent the recurrence of hypercalcemia.

## Figures and Tables

**Table 1.** Cancer Patients with Hypercalcemia in the OSCER Database

**Diagnosis**	**Number of Patients (Total 2 24 817)**	**Hypercalcemia (n)**	**Percentage (%)**
Lung cancer	27 618	1605	5.81
Breast cancer	73 458	1620	2.21
Colorectal cancer	23 574	560	2.37
Prostate cancer	8527	173	2.03
Renal cancer	2661	207	7.78
Other solid cancer	45 115	1571	3.48
Multiple myeloma	6328	736	11.63

OSCER, Oncology Services Comprehensive Electronic Records.

**Table 2.** Literature on Hypercalcemia in Renal Malignancies

**Author**	**No of Cases**	**Tumor Type**	**Bone Mets**	**PTHrP**	**Visceral Mets**	**Management of Tumor**	**Outcome of Hypercalcemia**	**Outcome of Tumor**
Pepper et al^19^	1	RCC	A	N	A	Partial nephrectomy	Controlled	Cured
Walther et al^17^	15	RCC	5/15	E in 3/3	15/15	Surgery	Controlled in 9/11	Progressed
Ueno et al^20^	1	Clear cell RCC	A	E	P	Surgery	Controlled	Progressed
Robertson et al^21^	1	RCC	A	U	P	Surgery	Uncontrolled	Progressed
Brereton et al^22^	1	RCC	A	N	P	Surgery, indomethacin	Controlled	Progressed
Musri et al^23^	1	SCC renal pelvis	A	U	P	Surgery, palliative	Temporary	Progressed
Gomes et al^24^	1	RCC	A	E	A	Chemotherapy, parathyroidectomy	Uncontrolled	Progressed
Karaca et al^18^	1	RCC	A	U	P	Surgery, sunitinib	Controlled	NA
Acikgoz et al^25^	1	SCC kidney	A	U	P	Cytoreductive surgery, chemo	Controlled	Progressed
Asao et al^26^	1	TCC renal pelvis	A	N	P	Nephrectomy, palliative chemo	Controlled	Progressed
Grubb et al^27^	1	TCC renal pelvis	A	U	A	Surgery	Controlled	Cured

**PTHrP, **parathyroid hormone-related peptide; RCC, renal cell carcinoma; SCC, squamous cell carcinomas; TCC, Transitional Cell carcinoma.

A, absent; P, present; U, undocumented; N, normal; NA, not available; E, elevated.

**Table 3.** Literature Available on Bladder Cancer with Hypercalcemia

**Author**	**No of Cases**	**Tumor Type**	**Bone Mets**	**PTHrP**	**Visceral Mets**	**Lymph Nodes Mets**	**Management of Tumor**	**Outcome of Hypercalcemia**	**Outcome of Tumor**	**Complications**
J K Benett^35^	4	TCC	A	U	A	A	Radiation, surgery	Controlled	Cured	None
De la Rosa^31^	1	TCC	A	U	A	A	Surgery	Controlled	Cured	None
A B Patel^36^	1	TCC	A	U	A	A	Surgery	Controlled	Cured	None
Richard Bringhurst^37^	1	TCC	A	U	A	P	Methotrexate	Temporary	Progressed	Renal failure, death
Chaudhary^28^	1	TCC	A	E	P	NA	Surgery	Temporary	Progressed	Death
Cesar V Reyes^34^	2	Small cell carcinoma	P	U	P	P	TURBT, laparotomy	Controlled	Progressed	Death
JE Kim^29^	2	TCC	P	E	P	P	Chemotherapy, palliative	Controlled	Progressed	Renal failure, fracture, obstruction, death
Sendur^33^	1	Small cell carcinoma	P	U	A	P	Surgery, chemotherapy, radiotherapy	Controlled	Progressed	None
Takashi Ando^32^	1	TCC	A	U	A	P	Supportive	Temporary	Progressed	Seizure, death
G Harel^30^	2	TCC	A	U	P	NA	Supportive	Controlled	Progressed	Death
Keiko Asao^38^	1	TCC (upper tract)	A	N	A	P	Surgery, supportive	Controlled	Progressed	Death
Yoshida T^39^	1	TCC	A	E	P	NA	Supportive	Uncontrolled	Progressed	Cardiac failure, renal failure, death
Hirakawa K^40^	3	TCC	A	U	P	NA	Supportive	NA	Progressed	Death

PTHrP, parathyroid hormone-related peptide.

A, absent; P, present; U, undocumented; N, normal; NA, not available; E, elevated.

**Table 4.** List of Reports on Penile Cancer with Hypercalcemia

**Author**	**No of Cases**	**Tumor Type**	**Bone Mets**	**PTHrP**	**Visceral Mets**	**Lymph Nodes Mets**	**Management of Tumor**	**Outcome of Hypercalcemia**	**Outcome of Tumor**	**Complications**
Dorfinger et al^41^	1	SCC	A	E	P	P	Surgery, chemotherapy	Temporary	Progressed	Death
E. Anderson et al^42^	1	SCC	A	U	A	P	Surgery	Resolved	Cured	None
Malakoff et al^43^	1	Epidermoid carcinoma	A	U	P	P	Surgery, radiotherapy, bleomycin	Temporary	Progressed	Death
Trejo Rosales et al^44^	1	SCC	A	E	A	NA	Palliative	Controlled	Progressed	Sepsis
Sardinas et al^45^	1	SCC	A	N	P	NA	Chemotherapy	Uncontrolled	Progressed	Death
Gandhi et al^46^	1	SCC	A	U	P	P	Surgery, chemotherapy, radiotherapy	NA	Progressed	Renal failure
R Kanta et al^47^	1	SCC	A	E	A	P	Supportive	Temporary	Progressed	Death
CC K Ho^48^	1	SCC	P	U	A	P	Supportive	Resolved	NA	Fracture

**PTHrP, **parathyroid hormone-related peptide; SCC, squamous cell carcinomas.

A, absent; P, present; U, undocumented; N, normal; NA, not available; E, elevated.

**Table 5.** Literature on Prostate Cancer with Hypercalcemia

**Author**	**No of Cases**	**Tumor Type**	**Bone Mets**	**PTHrP**	**Visceral Mets**	**Lymph Nodes Mets**	**Management of Tumor**	**Outcome of Hypercalcemia**	**Outcome of Tumor**	**Complications**
T Ando et al^50^	1	Neuroendocrine	P	E	P	P	Palliative	Uncontrolled	Progressed	Death
G Alhatemi et al^51^	1	Adenocarcinoma	P	N	A	NA	ADT, enzalutamide, docetaxel	Controlled	Progressed	Death
DC smith et al^52^	3	Neuroendocrine	P in 2/3	U	P	NA	Surgery	Controlled	Progressed	Renal failure, death

**PTHrP, **parathyroid hormone-related peptide; ADT, androgen deprivation therapy.

A, absent; P, present; U, undocumented; N, normal; NA, not available; E, elevated.

**Table 6.** Reports on Testicular Tumor with Hypercalcemia

**Author**	**No of Cases**	**Tumor Type**	**Bone Mets**	**PTHrP**	**Visceral Mets**	**Lymph Nodes Mets**	**Management of Tumor**	**Outcome of Hypercalcemia**	**Outcome of Tumor**	**Complications**
Grote et al^53^	1	Seminoma	A	U	A	NA	Surgery, chemotherapy	Controlled	Cured	None
MA da Silva et al^54^	4	Seminoma	A	U	A	NA	Surgery, chemotherapy	Controlled	Cured	None
Mac Diarmid et al^55^	1	Non seminoma	A	U	P	NA	NA	NA	NA	NA
Soescher et al^56^	1	Non seminoma	A	E	A	NA	Chemotherapy	Controlled	Cured	None
Rodríguez-Gutiérrez et al^57^	1	Seminoma	A	E	P	NA	Chemotherapy	Controlled	Cured	None

**PTHrP, **parathyroid hormone-related peptide.

A, absent; P, present; U, undocumented; N, normal; NA, not available; E, elevated.
